# Cognitive performance in older-age bipolar disorder: A cross-sectional study of anticholinergic load and clinical characteristics

**DOI:** 10.1192/j.eurpsy.2026.12247

**Published:** 2026-07-31

**Authors:** Simone Pardossi, Brisa Solé, Andrea Ruiz, Marta Bort, Arturo Rodríguez-Rey, Ainara Espluga, Meritxell González-Campos, Marc Valentí, Giovanna Fico, Andrea Murru, Jose Sanchez-Moreno, Anabel Martinez-Aran, Andrea Fagiolini, Eduard Vieta, Michele De Prisco, Laura Montejo

**Affiliations:** 1 Department of Molecular Medicine, School of Medicine, University of Siena, Siena, Italy; 2 Bipolar and Depressive Disorders Unit, Hospital Clinic de Barcelona, Barcelona, Spain; 3 Institut d’Investigacions Biomèdiques August Pi i Sunyer (IDIBAPS), Barcelona, Spain; 4 Departament de Medicina, Facultat de Medicina i Ciències de la Salut, Institut de Neurociències, Universitat de Barcelona (UBNeuro), Barcelona, Spain; 5 Sleep Disorders Unit, Hospital Clinic de Barcelona, Barcelona, Spain; 6 Centro de Investigación Biomédica en Red de Salud Mental (CIBERSAM), Instituto de Salud Carlos III, Madrid, Spain

**Keywords:** anticholinergic load, bipolar disorder, cognition, CRIDECO Anticholinergic Load Scale, defined daily dose, older-age bipolar disorder

## Abstract

**Background:**

Cognitive deficits are one of the core features of older-age bipolar disorder (OABD). Psychotropic medications may influence cognition, especially in older populations, where anticholinergic effects are relevant. This cross-sectional study aimed to investigate the potential impact of anticholinergic load on cognitive performance in individuals with OABD.

**Methods:**

One hundred and twenty-one euthymic or partially remitted OABD patients were included. Cognitive performance was assessed using a neuropsychological battery and summarized into attention, processing speed, working memory, visual and verbal memory, and executive functions. Anticholinergic load was assessed using the CRIDECO Anticholinergic Load Scale (CALS), and pharmacological burden was quantified using the defined daily dose (DDD). Associations were examined using hierarchical multivariable linear regression models, considering also relevant clinical covariates.

**Results:**

The interaction between CALS and DDD was associated with lower visual and verbal memory scores (*p* = 0.04). Age was inversely associated with cognitive performance across all domains (all *p* < 0.01). A higher number of manic episodes was associated with poorer attention, visual and verbal memory, and processing speed (all *p* < 0.01). Lifetime psychotic symptoms were associated with worse working memory (*p* = 0.02), and higher diazepam-equivalent doses were associated with worse visual and verbal memory performance (*p* = 0.02).

**Conclusions:**

Although anticholinergic medications may contribute to cognitive burden, their impact appears dose-related and limited compared with the broader influence of illness-related factors. In OABD patients, prioritizing the management of illness-related characteristics alongside careful pharmacological management may therefore be crucial for preserving cognitive functioning.

## Introduction

Bipolar disorder (BD) is a lifelong condition, and increasing life expectancy has led to a growing focus on older age bipolar disorder (OABD), a term used to describe individuals with BD aged ≥50 years old, according to the International Society for Bipolar Disorders (ISBD) OABD Task Force criteria [1]. One of the key aspects of this population is the higher risk of cognitive decline [2], as it is influenced by two converging factors: on the one hand, the aging process itself [3], and on the other hand, illness-related factors, which are intrinsically associated with cognitive symptoms [4]. Patients with OABD show a more impaired cognitive profile compared to age-matched individuals without BD and, in some studies, even compared to younger patients with BD [2]. This impairment mainly affects episodic memory, attention, processing speed, verbal fluency, and executive functions [2]. These deficits are also present during euthymia and often represent a major determinant of functional outcomes [5]. Data from the GAGE-BD consortium further highlight cognition as a central component of the OABD phenotype [6].

Cognitive performance in OABD is likely determined by the interaction of multiple factors [2, 7]. In addition to age, several clinical variables have been associated with cognitive outcomes, including manic episodes, psychotic symptoms, and educational level [2, 8]. Beyond these factors, and closely related to them, psychopharmacological treatment may represent a relevant contributor, although the available evidence remains scattered and heterogeneous [9, 10, 11] and is often not specifically investigated as a primary outcome in this field. In considering this aspect, it is important to emphasize that BD is a chronic, recurrent illness requiring long-term pharmacological treatment [12]. This also applies to OABD, in which pharmacotherapy represents one of the cornerstones of disease management [1]. On the one hand, pharmacological treatments, by stabilizing the illness, may indirectly improve cognitive functioning [13]. On the other hand, specific pharmacodynamic mechanisms may directly impact cognition [14–16]: anticholinergic burden is among the most plausible contributors, particularly in older individuals [17].

Acetylcholine plays a fundamental role in attention, learning, and memory, as it is involved in the encoding of new information and in the modulation of synaptic plasticity [18], facilitating afferent processing and reducing interference from previously stored representations, as well as supporting mechanisms such as persistent neuronal activity and long-term potentiation [18, 19]. Its disruption through muscarinic receptor blockade may negatively impact cognitive performance [18–20]. Recent evidence suggests a broader role of acetylcholine in its interaction with other neurotransmitter systems. In particular, it regulates learning-related plasticity by modulating dopaminergic signaling in a context- and time-dependent manner, effectively gating whether reinforcement signals lead to synaptic changes. Transient cholinergic pauses and bursts appear to shape striatal processing and determine the functional impact of dopamine on learning and behavior [21]. Studies conducted in community-dwelling older adults (aged ≥65–70 years) who were initially free of dementia or major cognitive impairment have shown that anticholinergic burden predicts worse cognitive performance over time across multiple domains, including global cognition, episodic memory, executive function, verbal fluency, and processing speed [22]. In patients with OABD, who, as individuals with BD, are frequently exposed to polypharmacy [23], and given that many psychotropic medications have anticholinergic properties [24], it is particularly important to assess the cumulative impact of these treatments using an aggregate measure of anticholinergic burden [25].

Several scales have been developed to quantify anticholinergic burden, including the Anticholinergic Cognitive Burden (ACB) scale [26]. These instruments aim to estimate the cumulative anticholinergic exposure derived from all medications taken by an individual, although they differ in the number of drugs included, scoring criteria, and clinical validation [26]. More recently, updated scales such as the CRIDECO Anticholinergic Load Scale (CALS) have been proposed. The CALS was developed through a systematic review of existing anticholinergic burden tools and pharmacological sources, and includes a broader list of medications with anticholinergic properties than previous scales, many of which are commonly prescribed psychotropic medications [24]. Each medication included in CALS is assigned a score according to its estimated anticholinergic potency, allowing the calculation of a cumulative anticholinergic load by summing the scores of all CALS-classified medications taken by each patient [24]. Both scales have already been used to investigate cognitive outcomes in psychiatric populations [27–29]. For example, a study including patients with schizophrenia, schizoaffective disorder, and bipolar disorder showed that higher CALS and ACB scores were associated with greater impairment in memory and working memory performance [29].

The aim of our study was to examine whether anticholinergic load was associated with domain-specific cognitive performance in euthymic or with subthreshold affective symptoms OABD individuals.

## Methods

### Study design

This cross-sectional observational study aimed to examine the association between anticholinergic load, pharmacological burden, and cognitive performance in clinically stable individuals with OABD. The reporting of this study was guided by the Strengthening the Reporting of Observational Studies in Epidemiology (STROBE) guidelines [30].

### Sample

The sample of patients included in this study is part of the OABD cohort recruited from the Bipolar and Depressive Disorders Unit at *Hospital* *Clínic* of Barcelona. Recruitment covered a time span from February 2006 to June 2024. The following inclusion criteria were adopted: (1) diagnosis of BD type I or II according to the Diagnostic and Statistical Manual of Mental Disorders Fourth Edition (DSM-4) [31] and DSM-5 [32]; (2) euthymic patients, defined as a score ≤ 6 on the Young Mania Rating Scale (YMRS) and a score ≤ 8 on the Hamilton Depression Rating Scale (HDRS) or patients in partial remission (HDRS ≤14 and YMRS ≤10); (3) being OABD, defined as age ≥ 50 years, according to the consensus of the ISBD Task Force [1]; (4) having complete data regarding pharmacological treatment; and (5) provision of written informed consent.

The exclusion criteria were: (1) estimated Intelligence Quotient <85; (2) presence of any central nervous system illness, other than a psychiatric disorder, that could interfere with neuropsychological performance (such as stroke, epilepsy, tumor, dementia, and multiple sclerosis); and (3) treatment with electroconvulsive therapy within the past 6 months.

This study was conducted in accordance with the ethical principles of the Declaration of Helsinki and Good Clinical Practice and was approved by the *Hospital* *Clínic* Ethics and Research Board. All participants provided written informed consent before inclusion in the study.

### Measures

A semi-structured interview was conducted to collect demographic (including age, sex, and years of education) and clinical variables, comprising BD types, the total number of mood episodes (manic, hypomanic, and depressive), and the number of each episode subtype. Additional variables included age at onset and type of onset (early vs. late), with late onset defined as a first episode occurring after the age of 50 years [33]. Information was also collected on the number of psychiatric admissions, duration of illness, history of suicidal ideation and suicide attempts, lifetime history of psychotic symptoms, somatic comorbidities, and pharmacological treatment. The presence and severity of any depressive and manic symptoms were evaluated using the HDRS [34, 35] and the YMRS [36, 37], respectively.

Pharmacological treatments being taken at the time of the cognitive assessment were considered. Neuropsychological assessments were performed at least 1 week after the last psychiatric visit. Moreover, given that patients were clinically stable, their pharmacological treatment remained unchanged. For each medication, the corresponding daily dosage was recorded. The treatment dose at which each drug was administered was converted into defined daily dose (DDD) equivalents, according to the World Health Organization (WHO) Collaborating Centre for Drug Statistics Methodology [38]. In addition, a cumulative DDD value was calculated for each patient by summing the DDDs of all concomitant medications. Within the WHO Anatomical Therapeutic Chemical (ATC)/DDD methodology [38], the DDD represents a standardized unit of drug consumption, allowing comparisons across different medications. Consistently, cumulative indices of drug burden have been proposed in other works [39, 40]. Moreover, the anticholinergic load of the pharmacological treatments was assessed using the CALS, which assigns a score to each drug according to its anticholinergic activity [24]. This scale classifies medications into different levels of anticholinergic potency and provides an estimate of the overall anticholinergic load associated with drug use. For each patient, the total anticholinergic load was calculated by summing the CALS scores of all medications taken. Medications not included in the CALS reference table were not assigned an anticholinergic score and were therefore classified as “not considered” for descriptive purposes. This classification should not be interpreted as evidence of absence of anticholinergic activity, but only as absence of classification within the CALS framework. Regarding benzodiazepines, all prescribed doses were converted into diazepam equivalents.

### Outcomes

The primary outcomes considered were the five cognitive domains: attention, processing speed, working memory, visual and verbal memory, and executive functions. To measure them, a comprehensive neuropsychological battery was administered. This battery was set following the recommendations of the ISBD cognitive assessment in bipolar disorder [41].

Attention was assessed using the Continuous Performance Test–II (CPT-II), version 5 [42], including omission errors, commission errors, and reaction time, as well as the Digit Span Forward subtest of the WAIS-III [43].

Processing speed comprised the Digit Symbol–Coding and Symbol Search subtests of the WAIS-III [44], together with Part A of the Trail Making Test (TMT-A) [45].

Working memory included the Arithmetic, Digit Span Backward, and Letter–Number Sequencing subtests of the WAIS-III [44].

Visual and verbal memory were evaluated using the California Verbal Learning Test (CVLT) [46], including total learning across trials, immediate free recall, delayed free recall, and the Rey–Osterrieth Complex Figure (ROCF) [47], using the immediate recall score.

Executive functions were assessed with the computerized Wisconsin Card Sorting Test (WCST) [48], using perseverative errors; the Stroop Color–Word Interference Test (SCWT) [49], using the interference score; Part B of the Trail Making Test (TMT-B) [45]; and phonemic (FAS) and semantic (animal naming) verbal fluency tasks from the Controlled Oral Word Association Test (COWAT) [50].

Cognitive composite scores were defined as the primary outcome measures. Patients’ raw test scores were converted into z-scores using the healthy control (HC) group as the reference (individuals aged over 50 years, without any psychiatric or neurological diagnosis, and with no first-degree family history of depression, bipolar disorder, or schizophrenia), matched for age and gender. Extreme z-values were winsorized at ±4. The z-scores obtained from tests in which higher scores indicate poorer performance (all scores of the CPT-II, WCST perseverative errors, and TMT-A and TMT-B) were reversed before calculating the composite scores. The visual and verbal memory domain was kept as a single composite score to maintain consistency with the domain-based approach adopted for the other cognitive outcomes and to avoid deriving a separate visual memory outcome from a single test score only. This approach is also consistent with previous work in OABD [7] and with methodological recommendations suggesting the use of composite cognitive measures rather than relying on individual neuropsychological tests when assessing cognition in BD [51]. Composite cognitive scores were computed for each cognitive domain by averaging the *z*-scores of the corresponding neuropsychological tests.

### Statistical analysis

Descriptive statistics were used to summarize sociodemographic and clinical characteristics, as well as psychotropic medication exposure. Continuous variables were reported as mean ± standard deviation (SD) when normally distributed, or as median (interquartile range, IQR) otherwise. Categorical variables were presented as *n* (%). Normality was assessed using the Shapiro–Wilk test.

Multivariable models were fitted separately for each cognitive domain. Only participants with complete data for all variables included in each model were considered; therefore, the sample size varied across models and cognitive domains. Drug exposure was modeled using total DDD, CALS total score, and their interaction term.

Three hierarchical models were tested:

- Minimally adjusted model: age, sex, DDD, CALS total score, and DDD × CALS interaction.
- Adjusted model: minimally adjusted model + diazepam-equivalent benzodiazepine dose.
- Fully adjusted model: adjusted model + number of manic episodes, number of depressive episodes, lifetime psychotic symptoms, and current HDRS score.

Model fit was quantified using *R* ^2^ for linear regression models. All tests were two-tailed, and statistical significance was set at *p* < 0.05. To assess the robustness of the main interaction finding in the fully adjusted model, we performed a nonparametric bootstrap analysis with 1,000 resamples, focusing on DDD × CALS interaction across cognitive domains. As an additional analysis, the fully adjusted models were repeated, including years of education as an additional covariate, given its established influence on cognitive performance [52]. Moreover, to address the reduction in sample size across hierarchical models, we additionally repeated the minimally adjusted and adjusted models using the same complete-case sample included in the fully adjusted model. These analyses were performed to distinguish the effect of adding covariates from the effect of changes in sample size across models. Multicollinearity in the fully adjusted models was assessed using variance inflation factors (VIFs).

Analyses were conducted in *R* version 4.5.3.

## Results

### Descriptive analysis

The characteristics of the study population are reported in Table 1. A total of 151 patients were initially considered, but 30 patients were excluded due to the lack of full pharmacological data, resulting in an analyzed sample of 121 patients (56.2% female, median age: 61.00 [57.00–67.00]). Of these, 61.2% had a diagnosis of BD type I, while 38.8% had BD type II. The enrolled patients were euthymic or presented residual symptoms, as reflected by the scores recorded on the HDRS (5.00 [3.00–8.00]) and YMRS (1.00 [0.00–4.00]).

**Table 1.** Sociodemographic, clinical, pharmacological, and cognitive characteristics Table 1. long description.

Variable	Levels	*n* (%) or mean ± SD or median [IQR]
Sociodemographic characteristics		
Age, years		61.00 [57.00–67.00]
Sex	Female	68 (56.2%)
	Male	53 (43.8%)
Occupational status	Unemployed	4 (4.5%)
	Temporary work leave	6 (6.7%)
	Permanent work leave	38 (42.7%)
	Retired	24 (27%)
	Working	15 (16.9%)
	Others	2 (2.2%)
Years of education		14.00 [10.75–17.00]
Clinical characteristics		
Diagnosis	Bipolar disorder type I	74 (61.2%)
	Bipolar disorder type II	47 (38.8%)
Age at illness onset, years		33.00 [23.00–41.50]
Hamilton depression rating scale total score		5.00 [3.00–8.00]
Young-mania rating scale total score		1.00 [0.00–4.00]
Illness duration, years		27.82 ± 12.71
Number of depressive episodes		5.00 [3.00–12.00]
Number of hypomanic episodes		3.00 [1.00–8.00]
Number of manic episodes		1.00 [0.00–2.00]
Total number of mood episodes		11.00 [6.00–23.25]
Number of psychiatric hospitalizations		1.00 [0.00–3.00]
Axis I comorbidity		11 (10.2%)
Axis II comorbidity		16 (15%)
Axis III comorbidity		81 (78.6%)
Type of first mood episode	Depressive	74 (74.7%)
	Hypomanic	6 (6.1%)
	Manic	19 (19.2%)
Psychotic symptoms at first episode (yes)		20 (21.3%)
Lifetime psychotic symptoms (yes)		53 (50.5%)
Pharmacological characteristics		
Lithium		58 (47.9%)
Anticonvulsants		82 (67.8%)
Antipsychotics		90 (74.4%)
Antidepressants		45 (37.2%)
Benzodiazepines		44 (36.4%)
CALS total score		3.00 [2.00–4.00]
Cognitive characteristics (*z*-scores)		
Attention		−0.70 ± 0.89
Processing speed		−1.34 ± 1.44
Working memory		−0.57 ± 1.01
Visual and verbal memory		−0.95 ± 1.24
Executive functions		−0.68 ± 0.89

Abbreviation: CALS, CRIDECO Anticholinergic Load Scale.

*Note:* Continuous variables are presented as mean ± standard deviation (SD) or median (interquartile range, IQR), as appropriate. Categorical variables are presented as number (percentage). Cognitive domain scores are expressed as z-scores relative to healthy controls aged >50 years, with lower scores indicating poorer cognitive performance. Percentages are calculated based on available data for each variable.

The median cumulative DDD was 2.00 [1.11–3.13]. Supplementary Table S1 shows the distribution of the different medications with psychiatric indications taken by patients in our study population (excluding benzodiazepines). In particular, for each drug, the value assigned according to the CALS is also reported [24]. Some medications taken by participants in our sample were not included in the CALS and were therefore labeled as “not considered.” The medications labeled as “not considered” in Supplementary Table S1 were agomelatine (*n* = 2, 1.7%), clotiapine (*n* = 5, 4.1%), lamotrigine (*n* = 38, 31.4%), pregabalin (*n* = 5, 4.1%), sulpiride (*n* = 1, 0.8%), topiramate (*n* = 7, 5.8%), and vortioxetine (*n* = 4, 3.3%). These medications were not included in the CALS and therefore did not contribute to the total CALS score.

Benzodiazepines are presented in Supplementary Table S2: 36.4% of the sample was receiving benzodiazepines, with a median dose of 10.00 mg of diazepam equivalents (10.00–20.00).

### Multivariable linear regression analyses

Within the visual and verbal memory domain, in the fully adjusted model (Table 2), the interaction between DDD and CALS was significant (*β* = −0.04, *p* = 0.04), with higher values of the interaction term being associated with lower scores. Bootstrap robustness analyses of the DDD × CALS interaction in the fully adjusted models are reported in Supplementary Table S3. Higher diazepam-equivalent doses were inversely associated with the visual and verbal memory score (*β* = −0.01, *p* = 0.02) as well. No statistically significant interactions between DDD and CALS were observed for the other cognitive domains.

**Table 2.** Fully adjusted model examining associations between pharmacological burden and cognitive performance across five domains Table 2. long description.

Predictor	*β*	SE	*t*-value	*p*
Attention (R^2^: 0.45, n = 75)				
Age	−0.02	0.00	−4.67	<**0.01**
Sex (female)	0.12	0.06	1.92	0.06
DDD	−0.01	0.02	−0.31	0.76
CALS total score	0.05	0.04	1.32	0.19
Diazepam equivalent mg	0.00	0.00	−0.24	0.81
Total number of manic episodes	−0.03	0.01	−4.17	<**0.01**
Total number of depressive episodes	−0.00	0.00	−2.06	**0.04**
Lifetime psychotic symptoms	0.10	0.06	1.64	0.11
HDRS score	−0.01	0.01	−0.80	0.42
Interaction DDD:CALS	0.00	0.01	−0.31	0.76
Executive functions (R^2^: 0.33, n = 77)				
Age	−0.02	0.00	−4.59	<**0.01**
Sex (female)	0.11	0.08	1.41	0.16
DDD	0.04	0.06	0.68	0.50
CALS total score	0.06	0.05	1.18	0.24
Diazepam equivalent mg	0.00	0.00	−0.36	0.72
Total number of manic episodes	−0.02	0.01	−1.62	0.11
Total number of depressive episodes	0.00	0.00	−1.37	0.17
Lifetime psychotic symptoms	0.01	0.08	0.18	0.86
HDRS score	−0.01	0.01	−0.59	0.56
Interaction DDD:CALS	−0.02	0.02	−1.20	0.24
Visual and verbal memory (R^2^: 0.47, n = 85)				
Age	−0.02	0.00	−5.60	<**0.01**
Sex (female)	0.12	0.07	1.63	0.11
DDD	0.07	0.05	1.21	0.23
CALS total score	0.08	0.04	1.81	0.08
Diazepam equivalent mg	−0.01	0.00	−2.44	**0.02**
Total number of manic episodes	−0.03	0.01	−3.03	<**0.01**
Total number of depressive episodes	0.00	0.00	1.20	0.23
Lifetime psychotic symptoms	−0.01	0.07	−0.20	0.84
HDRS score	0.01	0.01	1.00	0.32
Interaction DDD:CALS	−0.04	0.02	−2.13	**0.04**
Working memory (R^2^: 0.40, n = 74)				
Age	−0.01	0.00	−3.93	<**0.01**
Sex (female)	0.09	0.05	1.65	0.10
DDD	0.02	0.02	0.92	0.36
CALS total score	0.02	0.04	0.61	0.55
Diazepam equivalent mg	0.00	0.00	0.75	0.46
Total number of manic episodes	−0.01	0.01	−1.26	0.21
Total number of depressive episodes	0.00	0.00	0.98	0.33
Lifetime psychotic symptoms	−0.13	0.05	−2.38	**0.02**
HDRS score	−0.01	0.01	−1.61	0.11
Interaction DDD:CALS	−0.01	0.01	−1.17	0.25
Processing speed (R^2^: 0.56, n = 74)				
Age	−0.04	0.00	−7.25	<**0.01**
Sex (female)	0.10	0.09	1.16	0.25
DDD	0.00	0.03	−0.10	0.92
CALS total score	0.07	0.06	1.23	0.22
Diazepam equivalent mg	0.00	0.00	−0.38	0.70
Total number of manic episodes	−0.05	0.01	−3.51	<**0.01**
Total number of depressive episodes	0.00	0.00	−0.90	0.37
Lifetime psychotic symptoms	−0.07	0.09	−0.75	0.45
HDRS score	−0.01	0.01	−0.60	0.55
Interaction DDD:CALS	−0.01	0.02	−0.52	0.61

Abbreviations: CALS, CRIDECO Anticholinergic Load Scale; DDD, defined daily dose; HDRS, Hamilton Depression Rating Scale.

*Note:* Models were adjusted for age, sex, diazepam-equivalent dose, total number of manic episodes, total number of depressive episodes, lifetime psychotic symptoms, and depressive symptom severity (HDRS total score). Significant associations (*p* < 0.05) are shown in bold.

An additional fully adjusted model including years of education is reported in Supplementary Table S4. After inclusion of education years, the DDD × CALS interaction for visual and verbal memory showed a similar direction and magnitude, but no longer reached conventional statistical significance (*β* = −0.04, *p* = 0.06).

In the minimally adjusted model, the DDD × CALS interaction was significantly associated with visual and verbal memory performance (*β* = −0.05, *p* < 0.01), while no significant interaction was observed for the other cognitive domains (Supplementary Table S5). This association remained significant in the adjusted model (*β* = −0.05, *p* < 0.01; Supplementary Table S6). When the minimally adjusted and adjusted models were repeated using the complete-case sample of the fully adjusted model (Supplementary Tables S7 and S8), the DDD × CALS interaction remained significant for visual and verbal memory in both models (minimally adjusted model: *β* = −0.05, *p* = 0.02; adjusted model: *β* = −0.05, *p* < 0.01). In these restricted-sample models, the interaction was also significant for working memory (minimally adjusted model: *β* = −0.03, *p* = 0.03; adjusted model: *β* = −0.03, *p* = 0.04). However, after inclusion of clinical covariates in the fully adjusted model, the interaction remained statistically significant only for visual and verbal memory (*β* = −0.04, *p* = 0.04).

Across all three models, age was consistently and inversely associated with cognitive performance across all cognitive domains (all *p* < 0.01) (Table 2 and Supplementary Tables S5 and S6), indicating worse performance with increasing age. In the fully adjusted model, in addition to the effect of age across all cognitive domains (all *p* < 0.01; attention: *β* = −0.02; executive functions: *β* = −0.02; visual and verbal memory: *β* = −0.02; working memory: *β* = −0.01; and processing speed: *β* = −0.04), a higher number of manic episodes was negatively associated with cognitive performance across multiple domains, including attention (*β* = −0.03, *p* < 0.01), visual and verbal memory (*β* = −0.03, *p* < 0.01), and processing speed (*β* = −0.05, *p* < 0.01).

Finally, the presence of lifetime psychotic symptoms was associated with worse working memory performance (*β* = −0.13, *p* = 0.02).

Multicollinearity diagnostics did not indicate relevant collinearity in the fully adjusted models, with maximum VIF values ranging from 1.45 to 3.94 across cognitive domains.

## Discussion

The need to investigate the pharmacological aspects arises from both theoretical and practical perspectives: the relevance of psychopharmacology in the general context of BD and the clinical and therapeutic complexity of our population.

In our sample of patients, the fully adjusted model showed a significant impact of the interaction between anticholinergic load (CALS) and cumulative pharmacological burden (total DDD) only on the cognitive domain of visual and verbal memory. This result could be considered consistent with the functions mediated by the cholinergic neurotransmitter system, whose role is essential for episodic memory encoding and consolidation [18], and considering also that pharmacological models of cholinergic blockade impair the encoding and recall of newly learned information in episodic memory tasks [53]. However, some considerations should be made regarding this finding. First, the interaction between the two variables was significant, whereas the individual components were not. This pattern suggests that the observed association depends on the combined presence of anticholinergic properties and higher pharmacological exposure, rather than on either factor considered in isolation. Therefore, the association observed in visual and verbal memory may reflect an interplay between anticholinergic burden and overall pharmacological exposure. Moreover, when moving from the minimally adjusted to the more fully adjusted models, the significance of the interaction decreased as additional variables were included. Complete-case analyses suggested that this attenuation was not solely explained by the reduction in sample size, since the interaction remained evident for visual and verbal memory when the less adjusted models were restricted to the fully adjusted model sample. Although the bootstrap analysis yielded an estimate with the same direction and magnitude as the original model, it did not reach statistical significance. Finally, after accounting for education, the interaction between anticholinergic load and pharmacological burden in visual and verbal memory maintained a similar direction and magnitude but no longer reached conventional statistical significance. Taken together, these patterns may suggest a greater contribution of clinical factors, as also supported by the significant association between the number of lifetime manic episodes and performance in the same cognitive domain.

The lack of observed anticholinergic associations, except for memory domain, may be attributable to the multifactorial and heterogeneous nature of BD, in which cognitive functioning emerges from the interplay of numerous clinical, biological, and demographic determinants. Consistent with this framework, studies from the GAGE-BD consortium suggest that factors such as age, illness severity, and somatic burden show more consistent associations with cognition compared to global pharmacological measures [11]. In fact, the pharmacological treatments used in these patients represent a tool for the management of bipolar disorder itself and a means to achieve and maintain the euthymic state (or subsyndromal symptomatology) observed in the patients included in our sample. In this context, it cannot be excluded that the control of clinical factors already associated in the literature with cognitive functioning [8, 11], which are also partly significant in our regression models, may be related to relatively preserved cognitive profiles. However, the cross-sectional design of the study does not allow the exploration of possible indirect relationships between pharmacological treatment, clinical characteristics, and cognitive performance.

Another aspect to consider when dealing with pharmacological exposure is prescription or selection bias. Patients with more severe or complex clinical presentations might be more likely to receive more intensive and complex pharmacological regimens, resulting in higher overall DDD and potentially greater anticholinergic burden [54]. At the same time, illness severity itself may contribute to poorer cognitive performance, making it difficult to disentangle the potential cognitive effects of medication exposure from the impact of the underlying disease course [55]. For example, in previous evidence, prescription of anticonvulsants has been associated with markers of greater clinical complexity and different patterns of concomitant treatments [56]. Similarly, another study reported a better cognitive and clinical profile in OABD lithium users, explicitly discussing the impossibility of causal inference due to the cross-sectional design and the possibility of selection bias [57]. In particular, lithium requires monitoring and adherence, which may lead to its prescription being limited to patients considered able to comply with such requirements. In our study, patients were in euthymia or partial remission, and models were adjusted for clinical variables known to influence cognition, which may help mitigate, although not eliminate, this type of bias. In addition, given the cross-sectional design of the study, it is difficult to determine the long-term effects of pharmacological treatments.

The only factor that consistently emerged as significant and robust across all cognitive domains in all models was age: this finding is consistent with the idea that, in OABD, cognition may be influenced by a “double burden” effect: BD-related vulnerability may be combined with age-related decline [2]. Although the evidence is not entirely consistent, also due to factors that are not always measured [7], such as vascular burden, comorbidities, some domain-specific findings have been reported. In particular, the interaction between diagnosis and age has shown a steeper trajectory of decline in attention in BD patients than in healthy controls [8], especially for processing speed [58] and cognitive flexibility [59]. The fact that age emerges as a significant predictor in our study is clinically relevant also because BD is a condition potentially associated with accelerated biological aging [6]. This is particularly relevant considering that our sample already consisted of individuals aged over 50 years. Even within OABD, however, meaningful heterogeneity may persist. For instance, recent findings from the GAGE-BD project have shown that individuals aged 50–69 years and those aged ≥70 years differ in several clinical and somatic characteristics, supporting the idea that variability may remain across the later-life spectrum of BD [60].

Furthermore, a higher number of manic episodes was associated not only with poorer visual and verbal memory but also with worse performance in attention and processing speed, and the presence of lifetime psychotic symptoms with worse working memory. These findings are consistent with the limited literature available on OABD, suggesting that a greater recurrence of affective episodes, particularly manic episodes, is associated with a worse cognitive course in BD [7, 10]. Indeed, in the broader BD population, illness-course variables have also been shown to influence cognitive functioning [61]. The recurrence of affective episodes and symptom exacerbations has been proposed to contribute to neuroprogression [62], potentially leading to cumulative neurobiological changes over the course of the illness that could also impact cognition [63].

In addition to the anticholinergic mechanism, we also explored the potential impact of benzodiazepines, as previous reports using multivariable models including different pharmacological classes found that only benzodiazepines were significantly associated with cognitive outcomes [10]. Consistent with these findings, we observed that higher benzodiazepine doses, expressed as diazepam equivalents, were associated with poorer performance in visual and verbal memory.

Finally, the lack of significant associations between pharmacological variables and all the cognitive outcomes except visual and verbal memory should also be interpreted in light of the study’s limitations. First, as this is a cross-sectional study, it does not capture the temporal dimension of pharmacological exposure and instead provides only a snapshot at a given time point [64]. Some effects on cognition may occur rapidly, for example, with benzodiazepines, whose impact can emerge within hours or days of administration [65], and with antipsychotics, whose sedative effects are often more pronounced in the first weeks of treatment and may decrease over time [66]. Although evidence suggests that anticholinergic drugs are associated with an increased risk of cognitive decline and dementia with longer duration of exposure [67], their cognitive effects on domains such as memory and attention have been shown to occur rapidly in experimental studies [68]. Our analyses did not account for treatment duration, cumulative lifetime exposure, prior medication changes, or prior periods of exposure to anticholinergic drugs. Although some retrospective clinical information was available for some patients, reliable and systematic retrospective data on past pharmacological exposure were not available in the study database. This may have led to misclassification of the true long-term pharmacological and anticholinergic burden. Therefore, the present findings should be interpreted as reflecting the association between current pharmacological burden and cognitive performance, rather than the effect of cumulative lifetime exposure. Second, the sample size, although comparable to that of other studies in OABD, may have limited the statistical power to detect certain associations [69]. This is particularly relevant considering the wide range of variables that could potentially be included in regression models, which we had to restrict for this very reason. Third, the analyses were conducted post hoc: this may increase the likelihood of identifying statistically significant but potentially spurious associations and therefore require confirmation in future hypothesis-driven studies [70].

Fourth, although we adjusted our models for benzodiazepines, other pharmacodynamic mechanisms may also be involved. While anticholinergic effects are probably among the most relevant for cognition in older individuals, other mechanisms, such as antihistaminergic activity [71] or dopaminergic blockade [16, 72], may also contribute, for example, through sedation, reduced alertness, or effects on executive functions and processing speed. Fifth, some of the psychotropic medications used by participants did not have an assigned CALS value and were therefore not included in the calculation of the CALS total score, except for descriptive purposes. However, a possible anticholinergic effect of these medications, as well as a potential impact on cognition, cannot be excluded. Therefore, anticholinergic burden may have been underestimated in patients receiving drugs not considered by CALS. Nevertheless, the frequency of use of these medications in our sample was low, which likely limited their impact on the main findings.

In conclusion, in our sample of OABD individuals, anticholinergic load at the time of neuropsychological assessment was associated with visual and verbal memory performance only through its interaction with concurrent pharmacological burden, measured as total DDD. However, this association weakened when clinical variables reflecting illness severity were added to the models, although it remained statistically significant. In addition, markers of clinical severity, such as the number of manic episodes and lifetime psychotic symptoms, were associated with worse cognitive performance in our sample. These findings require confirmation in prospective studies specifically designed to further investigate the relationship between medications, symptomatic burden, and cognition.

Nevertheless, they should be interpreted in the context of the therapeutic role of pharmacological treatments, which are primarily used to control relapses and symptomatic exacerbations, which have themselves a clear impact on cognition. In this sense, our results suggest that the combination of overall dosage and anticholinergic burden may deserve attention, particularly with regard to visual and verbal memory. At the same time, given the broader impact of symptomatic indicators on other cognitive domains and the importance of preserving cognitive functioning in OABD individuals, preventing relapses or symptomatic exacerbations, including through pharmacological tools, should remain a main clinical priority. Beyond pharmacological strategies, preventing relapses through psychoeducation and considering cognitive and functional remediation interventions may also play a key role in preserving cognitive functioning in this population [51, 73].

## Supporting information

10.1192/j.eurpsy.2026.12247.sm001 Pardossi et al. supplementary material Pardossi et al. supplementary material

## Data Availability

The dataset used and analyzed in this study will be available upon reasonable request from the corresponding authors.

## Supplementary material

The supplementary material for this article can be found at http://doi.org/10.1192/j.eurpsy.2026.12247.

## Long descriptions

### Table 1 Long description

The table is organized into four main sections.

1. Sociodemographic characteristics

- Age: median 61.00 with I Q R 57.00 to 67.00.

- Sex: Female 68 (56.2 percent), Male 53 (43.8 percent).

- Occupational status: Permanent work leave is the most frequent at 38 (42.7 percent), followed by Retired at 24 (27 percent), Working at 15 (16.9 percent), Temporary work leave at 6 (6.7 percent), Unemployed at 4 (4.5 percent), and Others at 2 (2.2 percent).

- Years of education: median 14.00 with I Q R 10.75 to 17.00.

2. Clinical characteristics

- Diagnosis: Bipolar disorder type I 74 (61.2 percent), Bipolar disorder type I I 47 (38.8 percent).

- Age at illness onset: median 33.00 years.

- Hamilton depression rating scale total score: median 5.00.

- Young-mania rating scale total score: median 1.00.

- Illness duration: mean 27.82 plus or minus 12.71 years.

- Mood episodes: median total of 11.00, consisting of a median 5.00 depressive, 3.00 hypomanic, and 1.00 manic episodes.

- Psychiatric hospitalizations: median 1.00.

- Comorbidities: Axis I (10.2 percent), Axis I I (15 percent), Axis I I I (78.6 percent).

- First mood episode: Depressive (74 percent), Hypomanic (6 percent), Manic (19 percent).

- Psychotic symptoms: 21.3 percent at first episode, 50.5 percent lifetime.

3. Pharmacological characteristics

- Medications: Antipsychotics (74.4 percent), Anticonvulsants (67.8 percent), Lithium (47.9 percent), Antidepressants (37.2 percent), Benzodiazepines (36.4 percent).

- C A L S total score: median 3.00.

4. Cognitive characteristics (z-scores)

- Attention: minus 0.70 plus or minus 0.89.

- Processing speed: minus 1.34 plus or minus 1.44.

- Working memory: minus 0.57 plus or minus 1.01.

- Visual and verbal memory: minus 0.95 plus or minus 1.24.

- Executive functions: minus 0.68 plus or minus 0.89.

Navigate back to Table 1.

### Table 2 Long description

The table presents fully adjusted models for five cognitive domains. The columns are Predictor, beta, S E, t-value, and p.

1. Attention (R-squared 0.45, n = 75): Significant predictors include Age (beta -0.02, p < 0.01), Total number of manic episodes (beta -0.03, p < 0.01), and Total number of depressive episodes (beta -0.00, p = 0.04). Other predictors like D D D, C A L S total score, and Diazepam equivalent mg are non-significant.

2. Executive functions (R-squared 0.33, n = 77): The only significant predictor is Age (beta -0.02, p < 0.01).

3. Visual and verbal memory (R-squared 0.47, n = 85): Significant predictors include Age (beta -0.02, p < 0.01), Diazepam equivalent mg (beta -0.01, p = 0.02), Total number of manic episodes (beta -0.03, p < 0.01), and the Interaction D D D:C A L S (beta -0.04, p = 0.04).

4. Working memory (R-squared 0.40, n = 74): Significant predictors are Age (beta -0.01, p < 0.01) and Lifetime psychotic symptoms (beta -0.13, p = 0.02).

5. Processing speed (R-squared 0.56, n = 74): Significant predictors are Age (beta -0.04, p < 0.01) and Total number of manic episodes (beta -0.05, p < 0.01).

Abbreviations include C A L S (CRIDECO Anticholinergic Load Scale), D D D (defined daily dose), and H D R S (Hamilton Depression Rating Scale).

Navigate back to Table 2.
