# Supplementary material for: Cognitive performance in older-age bipolar disorder: A cross-sectional study of anticholinergic load and clinical characteristics
Source: Eur Psychiatry. 2026 Jul 31;69(1):e87. doi: 10.1192/j.eurpsy.2026.12247 (PMC13735821; doi:10.1192/j.eurpsy.2026.12247)
Supplement: Supplementary file 1 [file S0924933826122470sup001.docx]

**Cognitive Performance in Older-Age Bipolar Disorder: A Cross-Sectional Study of Anticholinergic Load and Clinical Characteristics**

**Simone Pardossi ^1,2,3^, Brisa Solé ^,3^, Andrea Ruiz ^2,3,4^, Marta Bort ^2,3,4^, Arturo Rodríguez-Rey^2,3,4^, Ainara Espluga ^2,3,4^, Meritxell González-Campos^2,3,4^, Marc Valentí ^2,3,4^, Giovanna Fico ^2,3,4^, Andrea Murru ^2,3,4,5^, Jose Sanchez-Moreno ^2,3,4,6^, Anabel Martinez-Aran ^2,3,4,6^*, Andrea Fagiolini^1^, Eduard Vieta ^2,3,4,6^ *, Michele De Prisco ^2,3,4,6 a^, Laura Montejo ^2,3,4,6 a^**

1 Department of Molecular Medicine, School of Medicine, University of Siena, 53100 Siena, Italy.

2 Bipolar and Depressive Disorders Unit, Hospital Clinic de Barcelona. c. Villarroel, 170, 08036 Barcelona, Spain

3 Institut d’Investigacions Biomèdiques August Pi i Sunyer (IDIBAPS), c. Villarroel, 170, 08036 Barcelona, Spain

4 Departament de Medicina, Facultat de Medicina i Ciències de la Salut, Institut de Neurociències, Universitat de Barcelona (UBNeuro), c. Casanova, 143, 08036 Barcelona, Spain

5 Sleep Disorders Unit, Hospital Clinic de Barcelona. C. Villaroel, 170, 08036 Barcelona, Spain

6 Centro de Investigación Biomédica en Red de Salud Mental (CIBERSAM), Instituto de Salud Carlos III, Madrid, Spain

**Corresponding Authors:**

Prof. Eduard Vieta, MD, PhD

Bipolar and Depressive Disorders Unit,

Institute of Neuroscience

IDIBAPS CIBERSAM

Hospital Clinic, University of Barcelona,

170 Villarroel St 12-0, 08036 Barcelona, Catalonia, Spain. Email: [evieta@clinic.cat](mailto:evieta@clinic.cat)

Dr. Anabel Martinez-Aran

Bipolar and Depressive Disorders Unit,

Institute of Neuroscience

IDIBAPS CIBERSAM

Hospital Clinic, University of Barcelona,

170 Villarroel St 12-0, 08036 Barcelona, Catalonia, Spain. Email: [AMARTIAR@clinic.cat](mailto:AMARTIAR@clinic.cat)

**Index**

[**Table S1. Distribution of psychotropic medications, CRIDECO Anticholinergic Load Scale (CALS), and defined daily dose (DDD) among users	4**](#_Toc235002917)

[**Table S2. Prevalence of benzodiazepine use in the total sample and diazepam-equivalent dose among users.	6**](#_Toc235002918)

[**Table S3. Bootstrap robustness analysis of the interaction between defined daily dose (DDD) and CRIDECO Anticholinergic Load Scale (CALS) in fully adjusted model	7**](#_Toc235002919)

[**Table S4. Fully adjusted model including education years	8**](#_Toc235002920)

[**Table S5. Linear regression models (Minimally adjusted model) examining associations between pharmacological burden and cognitive performance across five domains.	12**](#_Toc235002921)

[**Table S6. Linear regression models (Adjusted model) examining associations between pharmacological burden and cognitive performance across five domains.	13**](#_Toc235002922)

[**Table S7. Linear regression models (minimally adjusted model restricted to the fully adjusted model complete-case sample) examining associations between pharmacological burden and cognitive performance across five domains.	15**](#_Toc235002923)

[**Table S8. Linear regression models (adjusted model restricted to the fully adjusted model complete-case sample) examining associations between pharmacological burden and cognitive performance across five domains.	16**](#_Toc235002924)

# **Table S1. Distribution of psychotropic medications, CRIDECO Anticholinergic Load Scale (CALS), and defined daily dose (DDD) among users**

| Drug | CALS value | n (%) | DDD among users |
| --- | --- | --- | --- |
| Agomelatine | Not considered | 2 (1.7%) | 2.00 (2.00–2.00) |
| Aripiprazole | 1 | 14 (11.6%) | 0.50 (0.33–0.67) |
| Asenapine | 1 | 4 (3.3%) | 0.56 ± 0.31 |
| Bupropion | 1 | 5 (4.1%) | 1.00 ± 0.35 |
| Carbamazepine | 2 | 11 (9.1%) | 0.50 ± 0.23 |
| Clomipramine | 3 | 4 (3.3%) | 1.25 ± 0.75 |
| Clotiapine | Not considered | 5 (4.1%) | 1.00 (1.00–1.00) |
| Clozapine | 3 | 2 (1.7%) | 0.86 ± 0.71 |
| Desvenlafaxine | 1 | 2 (1.7%) | 2.00 (2.00–2.00) |
| Duloxetine | 0 | 3 (2.5%) | 1.50 (1.50–2.25) |
| Escitalopram | 1 | 7 (5.8%) | 1.89 ± 1.49 |
| Fluoxetine | 1 | 1 (0.8%) | 1.00 |
| Lamotrigine | Not considered | 38 (31.4%) | 0.67 (0.37–0.67) |
| Lithium | 1 | 58 (47.9%) | 0.89 (0.50–1.11) |
| Mirtazapine | 1 | 2 (1.7%) | 0.75 (0.62–0.88) |
| Olanzapine | 2 | 5 (4.1%) | 0.70 ± 0.33 |
| Oxcarbazepine | 2 | 5 (4.1%) | 0.69 ± 0.20 |
| Paliperidone | 1 | 3 (2.5%) | 0.81 ± 0.27 |
| Paroxetine | 2 | 1 (0.8%) | 1.50 |
| Perphenazine | 2 | 2 (1.7%) | 0.33 (0.30–0.37) |
| Phenelzine | 1 | 1 (0.8%) | 1.00 |
| Pramipexole | 1 | 1 (0.8%) | 0.33 |
| Pregabalin | Not considered | 5 (4.1%) | 0.25 (0.25–0.75) |
| Quetiapine | 2 | 63 (52.1%) | 0.50 (0.25–0.75) |
| Reboxetine | 0 | 1 (0.8%) | 0.50 (0.50–0.50) |
| Risperidone | 1 | 8 (6.6%) | 0.20 (0.10–0.67) |
| Sertraline | 1 | 7 (5.8%) | 2.00 (2.00–3.50) |
| Sulpiride | Not considered | 1 (0.8%) | 0.12 |
| Topiramate | Not considered | 7 (5.8%) | 0.58 (0.37–0.67) |
| Trazodone | 1 | 5 (4.1%) | 0.33 (0.17–0.33) |
| Valproate | 1 | 34 (28.1%) | 0.58 ± 0.21 |
| Venlafaxine | 1 | 14 (11.6%) | 1.50 (0.75–2.06) |
| Vortioxetine | Not considered | 4 (3.3%) | 1.00 (1.00–1.38) |
| Ziprasidone | 1 | 1 (0.8%) | 0.25 (0.25–0.25) |

| Table S2. Prevalence of benzodiazepine use in the total sample and diazepam-equivalent dose among users. | |
| --- | --- |
| Total Benzodiazepines | 44 (36.4%) |
|  |  |
| Clonazepam | 13 (10.7 %) |
| Diazepam | 8 (6.6%) |
| Lorazepam | 9 (7.4%) |
| Lormetazepam | 6 (5%) |
| Alprazolam | 3 (2.5%) |
| Clorazepato (dipotassium) | 2 (1.7%) |
| Bromazepam | 1 (0.8%) |
| Flurazepam | 1 (0.8%) |
| Diazepam equivalents mg of patients assuming Benzodiazepine | 10.00 (10.00–20.00) |

| Table S3. Bootstrap robustness analysis of the interaction between defined daily dose (DDD) and CRIDECO Anticholinergic Load Scale (CALS) in fully adjusted model | | | | | | | | |
| --- | --- | --- | --- | --- | --- | --- | --- | --- |
| Cognitive domain | R² | n | Original β | Original p | Bootstrap β | Bootstrap SE | Bootstrap 95% CI | Bootstrap p |
| Attention | 0.45 | 75 | 0.00 | 0.76 | -0.01 | 0.02 | [-0.04, 0.03] | 0.66 |
| Executive Functions | 0.33 | 77 | -0.02 | 0.24 | -0.02 | 0.03 | [-0.07, 0.02] | 0.39 |
| Visual and Verbal Memory | 0.47 | 85 | **-0.04** | **0.04** | -0.04 | 0.02 | [-0.08, 0.00] | 0.05 |
| Working Memory | 0.40 | 74 | -0.01 | 0.25 | -0.02 | 0.01 | [-0.05, 0.00] | 0.09 |
| Processing Speed | 0.56 | 74 | -0.01 | 0.61 | -0.01 | 0.03 | [-0.06, 0.04] | 0.69 |

# **Table S4. Fully adjusted model including education years**

| Predictor | β | SE | t value | p |
| --- | --- | --- | --- | --- |
| *Attention* **(R²: 0.49, n=75)** |  |  |  |  |
| Age | -0.02 | 0.00 | -5.22 | <0.01 |
| Sex(female) | 0.10 | 0.06 | 1.66 | 0.10 |
| DDD | -0.01 | 0.06 | -0.18 | 0.86 |
| CALS total score | 0.06 | 0.04 | 1.39 | 0.17 |
| Diazepam equivalent mg | 0.00 | 0.00 | -0.20 | 0.84 |
| Total number of manic episodes | -0.03 | 0.01 | -3.58 | <0.01 |
| Total number of depressive episodes | 0.00 | 0.00 | -2.39 | 0.02 |
| Lifetime psychotic symptoms | 0.08 | 0.06 | 1.33 | 0.19 |
| HDRS score | -0.01 | 0.01 | -1.51 | 0.14 |
| Education years | 0.03 | 0.01 | 2.75 | <0.01 |
| Interaction DDD:CALS | -0.01 | 0.02 | -0.27 | 0.79 |
| *Executive Functions* **(R²: 0.51, n=77)** | | | | |
| Age | -0.02 | 0.00 | -5.27 | <0.01 |
| Sex(female) | 0.10 | 0.07 | 1.46 | 0.15 |
| DDD | 0.01 | 0.06 | 0.23 | 0.82 |
| CALS total score | 0.06 | 0.05 | 1.31 | 0.19 |
| Diazepam equivalent mg | 0.00 | 0.00 | -0.35 | 0.73 |
| Total number of manic episodes | -0.01 | 0.01 | -1.04 | 0.30 |
| Total number of depressive episodes | 0.00 | 0.00 | -2.12 | 0.04 |
| Lifetime psychotic symptoms | 0.00 | 0.07 | -0.07 | 0.95 |
| HDRS score | -0.02 | 0.01 | -1.74 | 0.09 |
| Education years | 0.05 | 0.01 | 4.98 | <0.01 |
| Interaction DDD:CALS | -0.02 | 0.02 | -1.10 | 0.27 |
| Visual and Verbal Memory **(R²: 0.48, n=85)** | | | | |
| Age | -0.02 | 0.00 | -5.86 | <0.01 |
| Sex(female) | 0.11 | 0.07 | 1.61 | 0.11 |
| DDD | 0.06 | 0.06 | 0.96 | 0.34 |
| CALS total score | 0.09 | 0.05 | 1.93 | 0.06 |
| Diazepam equivalent mg | -0.01 | 0.00 | -2.54 | 0.01 |
| Total number of manic episodes | -0.03 | 0.01 | -2.83 | <0.01 |
| Total number of depressive episodes | 0.00 | 0.00 | 0.84 | 0.41 |
| Lifetime psychotic symptoms | -0.01 | 0.07 | -0.20 | 0.84 |
| HDRS score | 0.01 | 0.01 | 0.66 | 0.51 |
| Education years | 0.02 | 0.01 | 1.98 | 0.05 |
| Interaction DDD:CALS | -0.04 | 0.02 | -1.90 | 0.06 |
| *Working memory* **(R²: 0.45, n=74)** | | | | |
| Age | -0.01 | 0.00 | -4.01 | <0.01 |
| Sex(female) | 0.07 | 0.05 | 1.38 | 0.17 |
| DDD | 0.07 | 0.05 | 1.45 | 0.15 |
| CALS total score | 0.05 | 0.04 | 1.18 | 0.24 |
| Diazepam equivalent mg | 0.00 | 0.00 | 0.76 | 0.45 |
| Total number of manic episodes | -0.01 | 0.01 | -0.84 | 0.40 |
| Total number of depressive episodes | 0.00 | 0.00 | 0.79 | 0.43 |
| Lifetime psychotic symptoms | -0.11 | 0.05 | -2.14 | 0.04 |
| HDRS score | -0.02 | 0.01 | -1.94 | 0.06 |
| Education years | 0.02 | 0.01 | 2.22 | 0.03 |
| Interaction DDD:CALS | -0.03 | 0.01 | -1.76 | 0.08 |
| *Processing Speed* **(R²: 0.57, n=74)** | | | | |
| Age | -0.04 | 0.01 | -7.35 | <0.01 |
| Sex(female) | 0.09 | 0.09 | 1.04 | 0.30 |
| DDD | 0.01 | 0.08 | 0.07 | 0.94 |
| CALS total score | 0.08 | 0.06 | 1.23 | 0.22 |
| Diazepam equivalent mg | 0.00 | 0.00 | -0.26 | 0.80 |
| Total number of manic episodes | -0.04 | 0.01 | -3.10 | <0.01 |
| Total number of depressive episodes | 0.00 | 0.00 | -0.90 | 0.37 |
| Lifetime psychotic symptoms | -0.07 | 0.09 | -0.80 | 0.43 |
| HDRS score | -0.01 | 0.01 | -0.88 | 0.38 |
| Education years | 0.03 | 0.01 | 1.86 | 0.07 |
| Interaction DDD:CALS | -0.01 | 0.03 | -0.48 | 0.63 |

| Table S5. Linear regression models (Minimally adjusted model) examining associations between pharmacological burden and cognitive performance across five domains. | | | | |
| --- | --- | --- | --- | --- |
| Predictor | β | SE | t value | p |
| *Attention (R^2^: 0.22, n=95)* | | | | |
| Age | -0.02 | 0.00 | -4.25 | <0.01 |
| Sex (female) | 0.09 | 0.06 | 1.58 | 0.12 |
| DDD | -0.00 | 0.02 | -0.11 | 0.91 |
| CALS total score | 0.04 | 0.04 | 1.12 | 0.27 |
| Interaction DDD:CALS | -0.00 | 0.01 | -0.36 | 0.72 |
| *Executive functions (R^2^: 0.19, n=104)* | | | | |
| Age | -0.02 | 0.00 | -4.03 | <0.01 |
| Sex: female | 0.07 | 0.07 | 0.99 | 0.33 |
| DDD | 0.03 | 0.06 | 0.48 | 0.63 |
| CALS total score | 0.02 | 0.05 | 0.37 | 0.72 |
| Interaction DDD:CALS | -0.02 | 0.02 | -0.85 | 0.40 |
| *Visual verbal memory ( R^2^: 0.26, n=116)* | | | | |
| Age | -0.02 | 0.00 | -4.73 | <0.01 |
| Sex: female | 0.06 | 0.07 | 0.90 | 0.37 |
| DDD | 0.09 | 0.06 | 1.64 | 0.10 |
| CALS total score | 0.09 | 0.04 | 2.16 | 0.03 |
| Interaction DDD:CALS | -0.05 | 0.02 | -2.73 | <0.01 |
| *Working memory (R^2^: 0.19, n=104)* | | | | |
| Age | -0.01 | 0.00 | -4.16 | <0.01 |
| Sex: female | 0.08 | 0.06 | 1.50 | 0.14 |
| DDD | 0.02 | 0.02 | 1.05 | 0.30 |
| CALS total score | 0.03 | 0.04 | 0.85 | 0.40 |
| Interaction DDD:CALS | -0.02 | 0.01 | -1.46 | 0.15 |
| *Processing speed (R^2^: 0.35, n=104)* | | | | |
| Age | -0.03 | 0.01 | -6.76 | <0.01 |
| Sex: female | 0.11 | 0.08 | 1.31 | 0.19 |
| DDD | 0.01 | 0.03 | 0.42 | 0.67 |
| CALS total score | 0.07 | 0.05 | 1.30 | 0.20 |
| Interaction DDD:CALS | -0.02 | 0.02 | -1.48 | 0.14 |

# **Table S6. Linear regression models (Adjusted model) examining associations between pharmacological burden and cognitive performance across five domains.**

| Predictor | β | SE | t value | p |
| --- | --- | --- | --- | --- |
| *Attention (R^2^: 0.22, n=95)* |  |  |  |  |
| Age | -0.02 | 0.00 | -4.22 | <0.01 |
| Sex(female) | 0.09 | 0.06 | 1.57 | 0.12 |
| DDD | -0.00 | 0.02 | -0.11 | 0.91 |
| CALS total score | 0.04 | 0.04 | 1.11 | 0.27 |
| Diazepam equivalent mg | -0.00 | 0.00 | -0.03 | 0.98 |
| Interaction DDD:CALS | -0.00 | 0.01 | -0.35 | 0.72 |
| *Executive Functions* *(R^2^: 0.19, n=104)* | | | | |
| Age | -0.02 | 0.00 | -4.01 | <0.01 |
| Sex(female) | 0.07 | 0.07 | 0.99 | 0.33 |
| DDD | 0.03 | 0.06 | 0.48 | 0.63 |
| CALS total score | 0.02 | 0.05 | 0.37 | 0.71 |
| Diazepam equivalent mg | -0.00 | 0.00 | -0.09 | 0.93 |
| Interaction DDD:CALS | -0.02 | 0.02 | -0.85 | 0.40 |
| *Visual and Verbal Memory* *(R^2^: 0.26, n=116)* | | | | |
| Age | -0.02 | 0.00 | -4.75 | <0.01 |
| Sex(female) | 0.07 | 0.07 | 0.99 | 0.32 |
| DDD | 0.10 | 0.06 | 1.66 | 0.10 |
| CALS total score | 0.09 | 0.04 | 2.20 | 0.03 |
| Diazepam equivalent mg | -0.00 | 0.00 | -0.72 | 0.48 |
| Interaction DDD:CALS | -0.05 | 0.02 | -2.73 | <0.01 |
| *Working memory* *(R^2^: 0.19, n=104)* | | | | |
| Age | -0.01 | 0.00 | -4.15 | <0.01 |
| Sex(female) | 0.08 | 0.06 | 1.47 | 0.15 |
| DDD | 0.02 | 0.02 | 1.03 | 0.30 |
| CALS total score | 0.03 | 0.04 | 0.83 | 0.41 |
| Diazepam equivalent mg | 0.00 | 0.00 | 0.21 | 0.83 |
| Interaction DDD:CALS | -0.02 | 0.01 | -1.46 | 0.15 |
| *Processing Speed* *(R^2^: 0.35, n=104)* | | | | |
| Age | -0.03 | 0.01 | -6.72 | <0.01 |
| Sex(female) | 0.11 | 0.08 | 1.31 | 0.19 |
| DDD | 0.01 | 0.03 | 0.43 | 0.67 |
| CALS total score | 0.07 | 0.05 | 1.30 | 0.20 |
| Diazepam equivalent mg | -0.00 | 0.00 | -0.12 | 0.90 |
| Interaction DDD:CALS | -0.02 | 0.02 | -1.47 | 0.15 |

| Table S7. Linear regression models (minimally adjusted model restricted to the fully adjusted model complete-case sample) examining associations between pharmacological burden and cognitive performance across five domains. | | | | |
| --- | --- | --- | --- | --- |
| Predictor | β | SE | t value | p |
| *Attention* (R²: 0.22, n=75) | | | | |
| Age | -0.02 | 0.00 | -4.04 | <0.01 |
| Sex(female) | 0.09 | 0.07 | 1.23 | 0.22 |
| DDD | 0.01 | 0.07 | 0.17 | 0.87 |
| CALS total score | 0.04 | 0.05 | 0.90 | 0.37 |
| Interaction DDD:CALS | -0.01 | 0.02 | -0.48 | 0.63 |
| *Executive functions* (R²: 0.25, n=77) | | | | |
| Age | -0.02 | 0.00 | -4.34 | <0.01 |
| Sex(female) | 0.07 | 0.08 | 0.96 | 0.34 |
| DDD | 0.04 | 0.07 | 0.54 | 0.59 |
| CALS total score | 0.03 | 0.05 | 0.61 | 0.54 |
| Interaction DDD:CALS | -0.02 | 0.02 | -0.94 | 0.35 |
| *Visual verbal memory* (R²: 0.33, n=85) | | | | |
| Age | -0.02 | 0.00 | -5.50 | <0.01 |
| Sex(female) | 0.06 | 0.07 | 0.81 | 0.42 |
| DDD | 0.10 | 0.07 | 1.50 | 0.14 |
| CALS total score | 0.10 | 0.05 | 2.03 | 0.05 |
| Interaction DDD:CALS | -0.05 | 0.02 | -2.41 | 0.02 |
| *Working memory* (R²: 0.29, n=74) | | | | |
| Age | -0.01 | 0.00 | -4.01 | <0.01 |
| Sex(female) | 0.05 | 0.06 | 0.88 | 0.38 |
| DDD | 0.10 | 0.05 | 2.15 | 0.03 |
| CALS total score | 0.04 | 0.04 | 0.97 | 0.34 |
| Interaction DDD:CALS | -0.03 | 0.02 | -2.20 | 0.03 |
| *Processing speed* (R²: 0.41, n=74) | | | | |
| Age | -0.04 | 0.01 | -6.66 | <0.01 |
| Sex(female) | 0.03 | 0.10 | 0.31 | 0.76 |
| DDD | 0.06 | 0.08 | 0.70 | 0.49 |
| CALS total score | 0.07 | 0.07 | 1.10 | 0.28 |
| Interaction DDD:CALS | -0.03 | 0.03 | -0.97 | 0.34 |

# **Table S8. Linear regression models (adjusted model restricted to the fully adjusted model complete-case sample) examining associations between pharmacological burden and cognitive performance across five domains.**

| Predictor | β | SE | t value | p |
| --- | --- | --- | --- | --- |
| *Attention (R^2^: 0.22, n=75)* |  |  |  |  |
| Age | -0.02 | 0.00 | -4.04 | <0.01 |
| Sex(female) | 0.09 | 0.07 | 1.27 | 0.21 |
| DDD | 0.02 | 0.07 | 0.22 | 0.82 |
| CALS total score | 0.05 | 0.05 | 0.94 | 0.35 |
| Diazepam equivalent mg | -0.00 | 0.00 | -0.39 | 0.70 |
| Interaction DDD:CALS | -0.01 | 0.02 | -0.52 | 0.60 |
| *Executive Functions* *(R^2^: 0.25, n=77)* | | | | |
| Age | -0.02 | 0.00 | -4.35 | <0.01 |
| Sex(female) | 0.08 | 0.08 | 1.04 | 0.30 |
| DDD | 0.04 | 0.07 | 0.61 | 0.54 |
| CALS total score | 0.04 | 0.05 | 0.70 | 0.49 |
| Diazepam equivalent mg | -0.00 | 0.00 | -0.57 | 0.57 |
| Interaction DDD:CALS | -0.02 | 0.02 | -1.01 | 0.32 |
| *Visual and Verbal Memory* *(R^2^: 0.36, n=85)* | | | | |
| Age | -0.03 | 0.00 | -5.73 | <0.01 |
| Sex(female) | 0.08 | 0.07 | 1.09 | 0.28 |
| DDD | 0.12 | 0.07 | 1.75 | 0.08 |
| CALS total score | 0.11 | 0.05 | 2.31 | 0.02 |
| Diazepam equivalent mg | -0.00 | 0.00 | -1.85 | 0.07 |
| Interaction DDD:CALS | -0.05 | 0.02 | -2.68 | <0.01 |
| *Working memory* *(R^2^: 0.29, n=74)* | | | | |
| Age | -0.01 | 0.00 | -3.96 | <0.01 |
| Sex(female) | 0.05 | 0.06 | 0.81 | 0.42 |
| DDD | 0.10 | 0.05 | 2.06 | 0.04 |
| CALS total score | 0.04 | 0.04 | 0.89 | 0.37 |
| Diazepam equivalent mg | 0.00 | 0.00 | 0.46 | 0.65 |
| Interaction DDD:CALS | -0.03 | 0.02 | -2.11 | 0.04 |
| *Processing Speed* *(R^2^: 0.41, n=74)* | | | | |
| Age | -0.04 | 0.01 | -6.62 | <0.01 |
| Sex(female) | 0.03 | 0.10 | 0.33 | 0.75 |
| DDD | 0.06 | 0.08 | 0.71 | 0.48 |
| CALS total score | 0.08 | 0.07 | 1.11 | 0.27 |
| Diazepam equivalent mg | -0.00 | 0.00 | -0.21 | 0.83 |
| Interaction DDD:CALS | -0.03 | 0.03 | -0.98 | 0.33 |
